# Identification of the Tyrosine- and Phenylalanine-Derived Soluble Metabolomes of Sorghum

**DOI:** 10.3389/fpls.2021.714164

**Published:** 2021-09-14

**Authors:** Jeffrey P. Simpson, Jacob Olson, Brian Dilkes, Clint Chapple

**Affiliations:** ^1^Department of Biochemistry, Purdue University, West Lafayette, IN, United States; ^2^Purdue University Center for Plant Biology, West Lafayette, IN, United States

**Keywords:** sorghum, phenylpropanoids and phenolics, tyrosine and derivatives, metabol/nomics, specialized (secondary) metabolite

## Abstract

The synthesis of small organic molecules, known as specialized or secondary metabolites, is one mechanism by which plants resist and tolerate biotic and abiotic stress. Many specialized metabolites are derived from the aromatic amino acids phenylalanine (Phe) and tyrosine (Tyr). In addition, the improved characterization of compounds derived from these amino acids could inform strategies for developing crops with greater resilience and improved traits for the biorefinery. Sorghum and other grasses possess phenylalanine ammonia-lyase (PAL) enzymes that generate cinnamic acid from Phe and bifunctional phenylalanine/tyrosine ammonia-lyase (PTAL) enzymes that generate cinnamic acid and *p*-coumaric acid from Phe and Tyr, respectively. Cinnamic acid can, in turn, be converted into *p*-coumaric acid by cinnamate 4-hydroxylase. Thus, Phe and Tyr are both precursors of common downstream products. Not all derivatives of Phe and Tyr are shared, however, and each can act as a precursor for unique metabolites. In this study, ^13^C isotopic-labeled precursors and the recently developed Precursor of Origin Determination in Untargeted Metabolomics (PODIUM) mass spectrometry (MS) analytical pipeline were used to identify over 600 MS features derived from Phe and Tyr in sorghum. These features comprised 20% of the MS signal collected by reverse-phase chromatography and detected through negative-ionization. Ninety percent of the labeled mass features were derived from both Phe and Tyr, although the proportional contribution of each precursor varied. In addition, the relative incorporation of Phe and Tyr varied between metabolites and tissues, suggesting the existence of multiple pools of *p*-coumaric acid that are fed by the two amino acids. Furthermore, Phe incorporation was greater for many known hydroxycinnamate esters and flavonoid glycosides. In contrast, mass features derived exclusively from Tyr were the most abundant in every tissue. The Phe- and Tyr-derived metabolite library was also utilized to retrospectively annotate soluble MS features in two *brown midrib* mutants (*bmr6* and *bmr12*) identifying several MS features that change significantly in each mutant.

## Introduction

*Sorghum bicolor* (L.) is a C4 cereal crop known for its adaptation to, and exceptional productivity in, hot and water-limited environments. In addition to being a food and forage crop, especially in arid climates, there is interest in improving the properties of sorghum for the biorefinery (Brenton et al., 2016). Sorghum is genetically diverse and has a high potential for genetic improvement through breeding (Brenton et al., 2016). One potential path for improvement is the formulation of new or modified profiles of metabolites to enhance the resilience of the plant to biotic and abiotic stress.

Untargeted mass spectrometry (MS) can detect and quantify many of the metabolites contained in plant tissues. The output of these analyses is a list of mass features, each associated with a mass to charge ratio (m/z), column retention time, and ion count. The term mass feature rather than compound is appropriate because features can include the parental ions of true metabolites and in-source-generated molecular fragments or adducts. Untargeted MS datasets are rich in information concerning the metabolic repertoires of plants but are insufficient to predict compound structures. Although some metabolites can be identified through comparison to authentic standards and interpretation of fragment ions generated by higher-order MS, in untargeted MS, there are still hundreds to thousands of “unknown” metabolite features that have no structural information associated with them.

To increase the informational value of untargeted MS datasets we developed an R-based program called “Precursor of Origin Determination in Untargeted Metabolomics” (PODIUM) (Simpson et al., 2021). In this procedure, a heavy mass isotope representing a precursor to a class of metabolites (e.g., ring labeled ^13^C_6_ phenylalanine (Phe) to identify phenylpropanoids) is fed to plants, and MS features that incorporate the isotope are identified from mass spectrometry data that was processed by XCMS (Smith et al., 2006). The labeled and unlabeled features are then annotated as co-eluting isotopologues and the natural abundance isotopolog (i.e., light or ^12^C_6_) is identified. PODIUM was first applied to identify Phe-derived MS features produced in stems of wild-type *Arabidopsis* and 10 genotypes with defects in phenylpropanoid metabolism (Simpson et al., 2021). Over 2,000 MS features, which account for almost 30% of the negative-ion signals collected, were Phe-derived across the different genotypes. This library of metabolites produced from Phe was then used to annotate Phe-derived mass features within an untargeted metabolome of 440 *Arabidopsis* ecotypes. Genome-wide association mapping of those Phe-derived metabolites uncovered multiple loci contributing to natural variation in phenylpropanoid metabolism.

**Figure 1:**
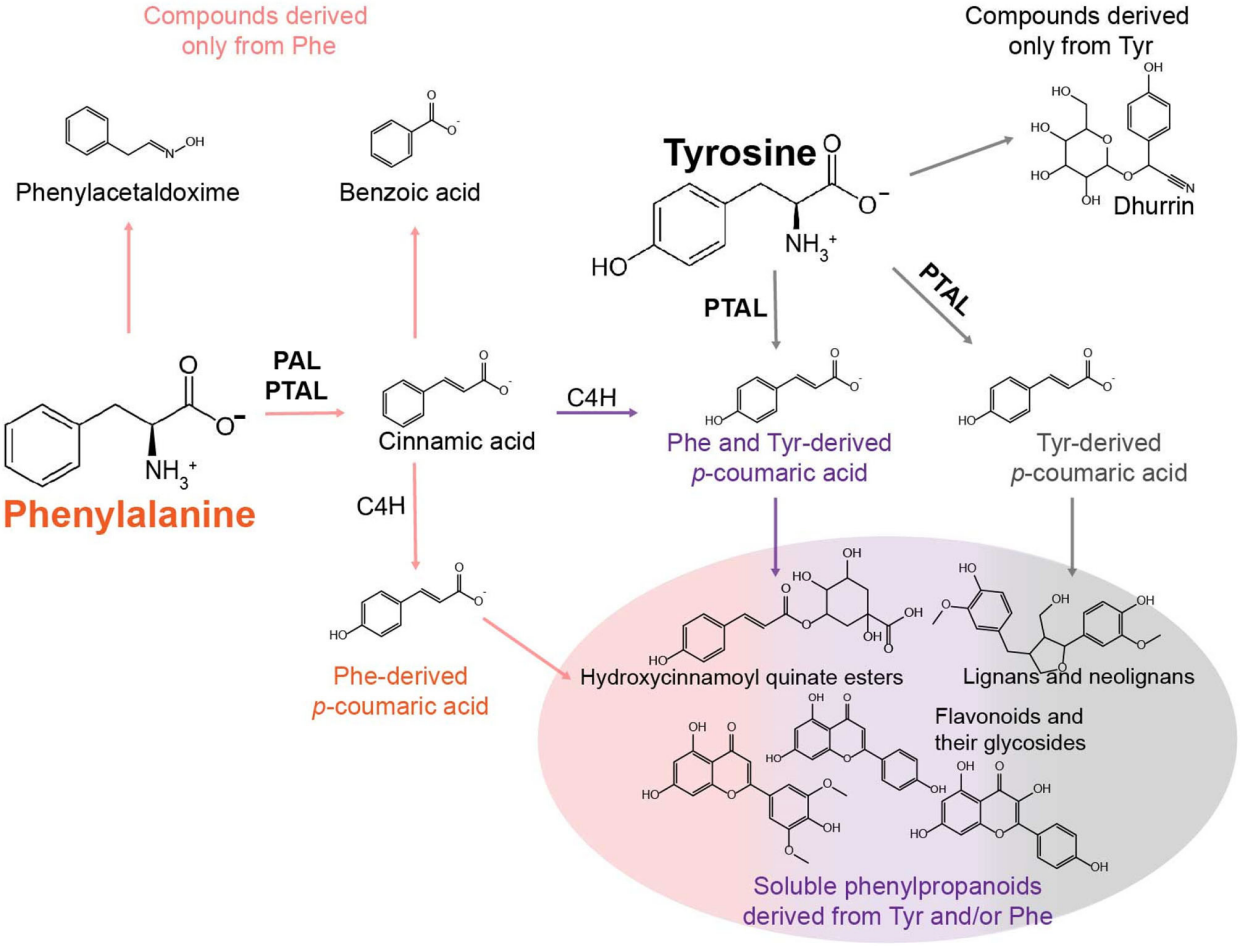
Model describing some of the metabolic fates of Phe and Tyr in phenylalanine/tyrosine ammonia-lyase (PTAL)-containing grasses. The primary fate of Phe is conversion to cinnamic acid by phenylalanine ammonia-lyase (PAL) or PTAL. Cinnamic acid can then be hydroxylated into *p-*coumarate by cinnamate 4-hydroylase (C4H) or converted to metabolites that are not *p*-hydroxylated (e.g., benzoic acid). Phe may also enter other pathways devoted to the production of phenylacetic acid via phenylacetaldoxime or phenylethanol. In sorghum, the primary fates of Tyr are the conversion into *p*-coumaric acid by PTAL or metabolism to the cyanogenic glycoside dhurrin. The different colored arrows leading to and from *p*-coumaric acid structures are meant to illustrate the multiple *p*-coumaric acid pools that can be derived from Phe via PAL, Phe via PTAL (orange), Phe and Tyr via PTAL (purple), and Tyr via PTAL (gray). Each of these pools may be utilized in different ways to produce downstream soluble phenylpropanoids.

The objective of this study was to catalog specialized metabolites in sorghum derived from the aromatic amino acids Phe and Tyr. Phe and Tyr provide the core structure to a variety of ubiquitous, lineage-, and species-specific specialized metabolites in plants, including soluble phenylpropanoids, lignin, and cyanogenic glycosides, which are compounds that are particularly relevant to this study (Widhalm and Dudareva, 2015; Tohge and Fernie, 2017; Schenck and Maeda, 2018). All plants synthesize phenylpropanoids from Phe via Phe ammonia lyase (PAL), but grasses can also produce phenylpropanoids from Tyr through the Tyr ammonia-lyase (TAL) activity of some PAL enzymes. Thus, this bifunctional enzyme, called Phe/Tyr ammonia-lyase (PTAL), can deaminate Phe and Tyr into cinnamic acid and *p*-coumaric acid, respectively (Barros and Dixon, 2020). In the Phe-derived pathway, cinnamic acid can be hydroxylated by cinnamate 4-hydroxylase (C4H) to generate *p*-coumaric acid, which could combine into a pool of *p*-coumaric acid that is derived from the TAL activity of PTAL (Figure 1). However, in the model grass *Brachypodium*, Barros et al. (2016) identified differences in the proportions of lignin and other phenylpropanoids derived from Phe or Tyr and through PAL or PTAL, suggesting that the pools of Phe- and Tyr-derived *p-*coumaric acid are not entirely shared or equivalent and that each pool contributes to different end products.

In this study, PODIUM was applied to identify Phe- and Tyr-derived metabolomes (FDM and YDM, respectively), in three tissues of the sorghum genotype BTx623. Ring-labeled ^13^C_6_-Phe and ^13^C_6_-Tyr were fed independently to sorghum seedlings, and metabolite features derived from these amino acids were then identified in the untargeted liquid chromatography-MS (LC-MS) dataset. These data allowed us to estimate the relative incorporation of labeled Phe and Tyr into features labeled with both precursors. Finally, the FDM and YDM were also annotated in two brown midrib mutants (*bmr6* and *bmr12*) that have defects in the phenylpropanoid pathway enzymes cinnamyl alcohol dehydrogenase (CAD) and caffeic acid/5-hydroxyferulic acid *O*-methyltransferase (COMT), respectively.

## Methods

### Plant Material and Growth Conditions

*S. bicolor* (L.) (BTx623 genotype) plants used for Phe and Tyr feeding were grown in Redi-Earth Plug and Seedling Mixture (Sun Gro Horticulture, Quincy MI) augmented with Scotts Osmocote Plus controlled-release fertilizer (Hummert International, Earth City, MO). Plants were grown to their three leaf stage (~10 days post emergence) in a greenhouse under long-day conditions [25°C/23°C (day/night) 16 h light/8 h dark photoperiod] with natural light supplemented with high-pressure sodium lamps (Philips, MASTER GreenPower T400W, Belgium). The *bmr6* and *bmr12* mutants and their wild-type (BTx623) controls were grown under the same conditions.

### Phenylalanine and Tyrosine Feeding

Phe and Tyr feeding was performed similarly to the methods of Wang P. et al. (2018) and Simpson et al. (2021). Plants were removed from the soil, washed with water, and placed into a 120 ml beaker containing the labeling medium along with two other replicate plants. The labeling medium consisted of 30 ml of a Murashige and Skoog medium supplemented with either 1 mM L-phenylalanine (Sigma) or L-tyrosine, or 1 mM ring-[^13^C_6_]-labeled L-phenylalanine or ring-[^13^C_6_]-labeled L-tyrosine [Cambridge Isotope Laboratories, Tewskbury, MA. Cat No. CLM-1055 (Phe) or CLM-1542 (Tyr)]. Plants were fed for 24 h in the greenhouse under the conditions described above, after which, each plant was rinsed with water and patted dry. The roots, basal 2 cm of the plant (representing the stem and 2 cm of leaf sheath and developing leaves), and 5 cm of the non-fully expanded leaves were dissected from the plant, flash-frozen in liquid nitrogen, and stored at -70°C until metabolite extraction.

### Metabolite Extraction and LC-MS Analysis of Soluble Metabolites

Soluble metabolites were extracted from frozen tissue in 50% aqueous methanol (v/v) at a concentration of 100 mg fresh mass ml^−1^ at 65°C for 2 h, with vortexing every 30 min. Samples were then centrifuged for 5 min at 13,000 × *g*, and the soluble fraction was transferred to a new tube. Samples were concentrated in a speed vacuum at 30°C and the dried extract was then re-dissolved in 50% aqueous methanol (v/v) at 20% of the original volume. All extracts were stored at −20°C prior to LC-MS analysis.

Chromatographic separations were performed using an Agilent 1100 HPLC system (Agilent Technologies, Palo Alto, CA, USA) with a Shimadzu Shim-pack XR-ODS (3 × 75 mm × 2.2 μm, Shimadzu Corp., Japan) separation column and a 5 μl injection volume. The LC and MS running conditions are described in Simpson et al. (2021). Following data collection, Agilent MassHunter raw data (.d directories) were converted to mzXML using the msconvert tool in ProteoWizard. The mzXML files were grouped into different folders based on tissue and treatment and processed with PODIUM (available at https://github.com/chapple-lab/podium) or XCMS (Smith et al., 2006) for the unfed *bmr6, bmr12*, and wild-type samples. Peak picking was performed using centWave using the following parameters: ppm = 15, peakwidth = c (3, 27), snthresh = 15, and prefilter = c (2, 15). Peaks were aligned using “obiwarp,” grouped with bw = 2, mzwid = 0.02, and minsamp = 3, and fillChromPeaks was used to integrate peaks across all samples that were not detected during the initial peak picking.

## Results

### Phenylalanine and Tyrosine Are Used to Produce Unique and Common MS Features

The FDM and YDM libraries were established on three leaf stage greenhouse-grown sorghum. Plants from the inbred line BTx623 were fed ^13^C_6_ ring-labeled Phe and Tyr by removing whole seedlings from their pots and placing them in beakers for 24 h with enough ^12^C or ^13^C_6_-Phe or ^13^C_6_-Tyr feeding solution to cover the roots. Following feeding, plants were harvested into three portions: (1) roots, (2) the 2 cm section above the roots encompassing the stem and portions of developing leaves and leaf bases (called “base” here), and (3) a 5 cm section above the base containing developing and unexpanded leaf blades and leaf sheaths (called “leaf” here). Semi-polar metabolites were extracted from each sample and analyzed by LC-MS using reverse-phase chromatography and negative-mode ionization. The resulting MS data were run through the XCMS-based PODIUM pipeline (Smith et al., 2006; Simpson et al., 2021) to identify the FDM and YDM. Only MS features that eluted between 100 and 1,100 s in the LC-MS gradient were included in this FDM and YDM analysis, and labeled and unlabeled Phe and Tyr were removed from downstream analyses.

**Figure 2:**
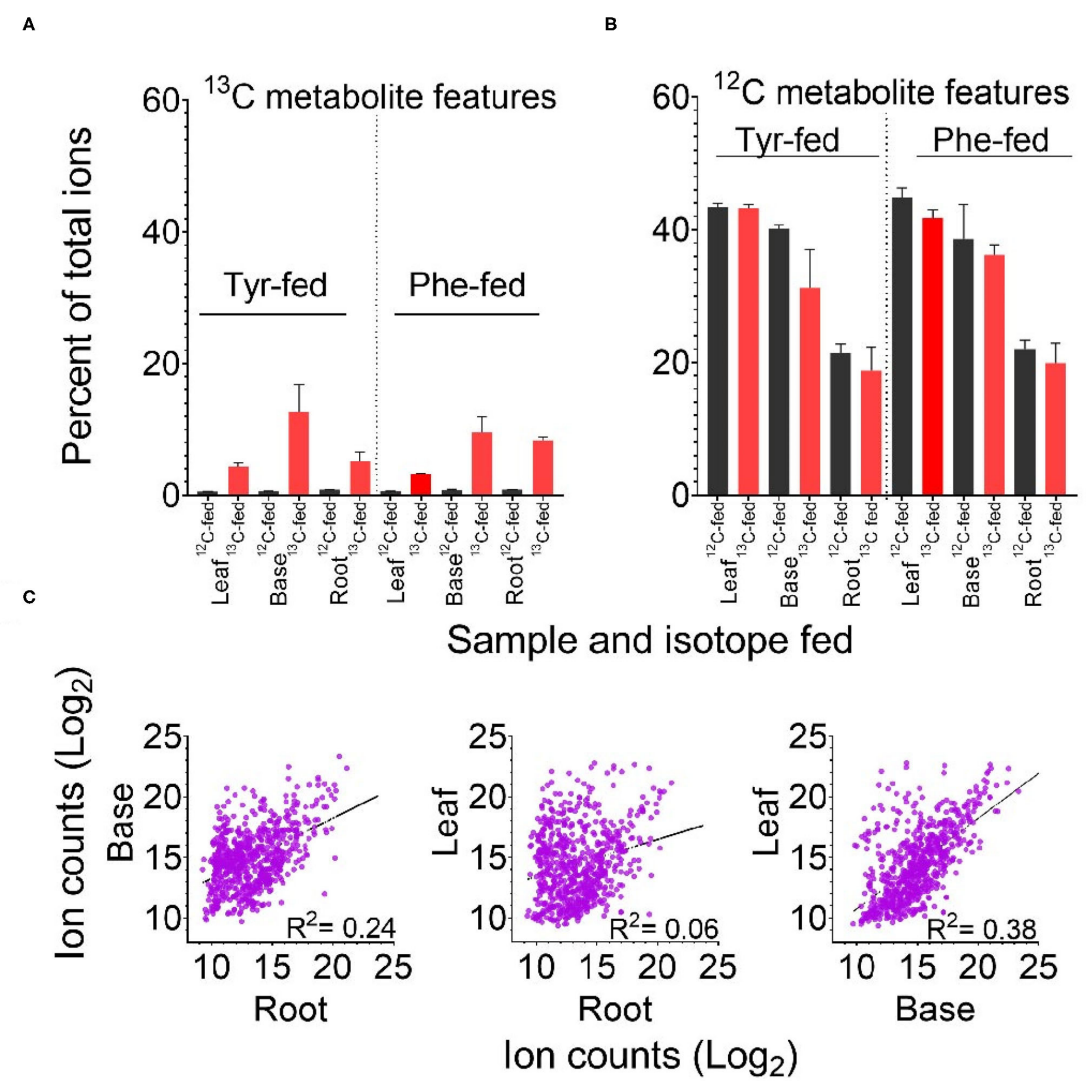
Labeling of predicted Tyr- and Phe-derived metabolite features according to feeding regime and tissue. **(A)** Percent of the total ion count in metabolite features predicted to be derived from Tyr or Phe. **(B)** Percent of the total ion count in ^13^C labeled features predicted to be derived from ^13^C Tyr or ^13^C Phe. Error bars in panels A and B indicate +/– SD of three biological replicates. **(C)** Comparison of log_2_ ion counts in predicted Phe- and Tyr-derived metabolite features (^12^C containing in ^12^C Phe-fed and ^12^C Tyr-fed tissue) across tissues. The broken line in each graph represents the best fit from linear regression and the correlation coefficient (*R*^2^) is reported.

In total, the PODIUM pipeline identified 668 metabolite features containing one to three phenyl rings derived from Phe or Tyr. The ^13^C_6_-containing metabolite features derived from Phe or Tyr represented 5–13% of the total ion counts in ^13^C-fed samples (Figure 2A). In some cases, the signal derived from a mass feature with a +6, +12, or +18 mass in the samples that were fed with the ^12^C isotope represented either co-chromatographing features and/or a signal value derived from the “peak-filling” step employed by XCMS integration. As expected for *bona fide* metabolite features, but not for such artifacts, the majority of features identified by PODIUM were more abundant in the heavy-isotope label fed samples. Of the three samples, the base- and root-derived extracts incorporated the most ^13^C-Phe and -Tyr, which may reflect better uptake of the label due to the closer proximity to the feeding solution.

Identification of these Phe- and Tyr-derived features in the ^12^C-fed samples allowed us to calculate the contribution of these two precursors. Taken together, the FDM and YDM were between 20 and 45% of the total ion count across the three tissues (Figure 2B). Aggregated ion counts for predicted Phe- and Tyr-derived metabolite features in the base and leaf portions were similar, while the ion signal from root samples was approximately 50% lower. There was no linear relationship between the abundance of specific Phe- and Tyr-derived ^12^C metabolite features between tissues, indicating that each tissue produces distinct metabolite profiles (Figure 2C).

**Figure 3:**
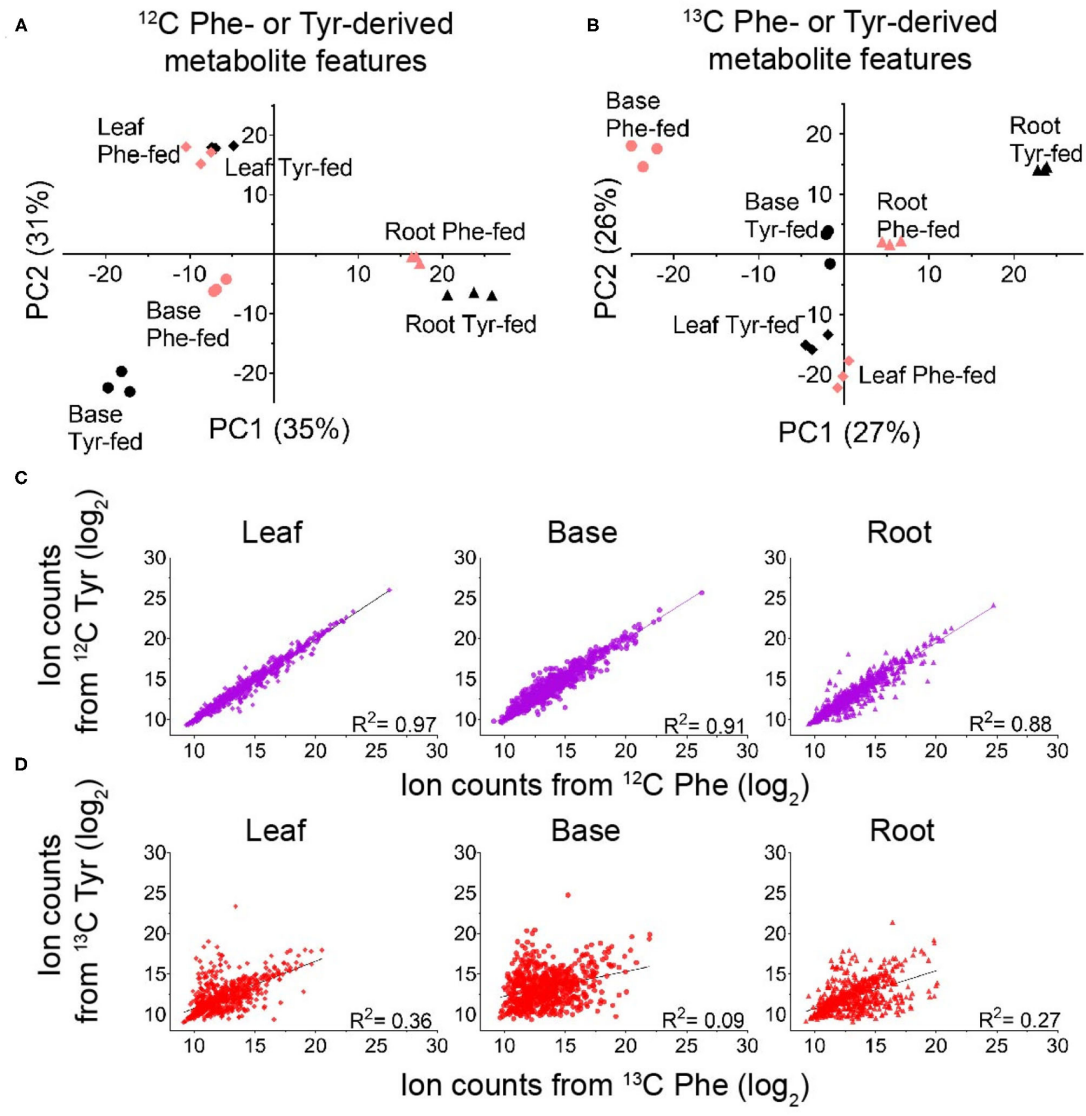
Identifying global differences in Phe- and Tyr-derived metabolite features between labeling regimes. **(A,B)** Principal component analysis (PCA) score plots showing the effect of genotype on the accumulation of metabolite features derived from ^12^C Phe and ^12^C Tyr **(A)** or ^13^C Phe and ^13^C Tyr **(B)** (red, Phe-labeled; black, Tyr-labeled) (*n* = 3). **(C,D)** Log_2_ ion counts for Phe- and Tyr-derived metabolite features compared between labeling treatments. **(C)** Log_2_ ion counts in ^12^C-containing metabolite features in samples fed with ^12^C Phe (x-axis) and samples fed with ^12^C Tyr (y-axis). **(D)** Log_2_ ion counts in ^13^C containing metabolite features in samples fed with ^13^C Phe (x-axis) and ^13^C Tyr (y-axis). The broken line in each graph represents the best fit from linear regression and the correlation coefficient is reported in each graph.

Replicated samples from the ^12^C-fed plants grouped with each other along the principal components, whereas replicated samples from the different tissues were separated (Figure 3A). Some effects of precursor supplementation were also visible in these plots. Notably, within the root and base tissues, Phe- and Tyr-fed samples were separated in the score plot (Figure 3A), indicating that the administration of Phe, Tyr, or both altered the synthesis of some ^12^C features in their respective FDM's and YDM's. However, comparing the accumulation of individual ^12^C-containing features between the Phe-fed and Tyr-fed samples by linear regression revealed a very strong linear relationship between most metabolite features (Figure 3C). This demonstrates that the administration of the ^12^C amino acids had only a minor effect on the abundance of ^12^C metabolites in the FDM and YDM.

Unlike the analysis of the ^12^C features, principal component analysis (PCA) and linear regression using the abundances of the ^13^C_6,12,18_-containing features is a direct measure of the *de novo* production of metabolites during the 24 h feeding. Thus, this analysis can detect metabolites produced from Phe and Tyr via PTAL activity and identify the features that are differentially or uniquely derived from either amino acid. Principal components calculated from these data showed clear separation of the root, base, and leaf samples (Figure 3B). Heavy isotope-labeled Phe or Tyr feeding also resulted in clear separation for all three tissues (Figure 3B). Taken together, this suggested that Tyr and Phe feeding had different effects on compound *de novo* biosynthesis (Figure 3B). Linear regression comparing the abundances of the ^13^C metabolite features between feedings also demonstrated the presence of some metabolites derived primarily from either Phe or Tyr, although the majority were of mixed origin (Figure 3D). Most metabolite features were labeled from both Phe and Tyr, indicating that sorghum employs both precursors to synthesize many of its soluble phenyl-ring-containing metabolites. The existence of metabolite features that fall closer to each axis (Figure 3D) indicates that some PAL or PTAL-dependent metabolites and were preferentially derived from one amino acid.

### Phe- and Tyr-Derived Metabolites Have Distinct Patterns of ^13^C Incorporation, Chromatographic Properties, and Abundances

Tyr and Phe can be metabolized into phenyl ring-containing nitrogenous metabolites or compounds resulting from amino group removal by transamination, oxidative deamination, or, most prominently, deamination through the ammonia-lyase activity of PAL or PTAL (Widhalm and Dudareva, 2015; Schenck and Maeda, 2018). Some of these metabolite pathways that utilize Phe and Tyr are uniquely fed by one amino acid (i.e., Tyr-derived cyanogenic glycosides, Phe-derived cinnamoyl, or phenylacetaldoxime derivatives). In grasses, both amino acids can act as precursors for phenylpropanoids produced downstream of *p-*coumaric acid. In this study, the incorporation of labeled Phe and Tyr into the FDM and YDM were compared to estimate the relative contribution of Phe and Tyr into metabolites derived through PAL and/or PTAL.

Label incorporation into metabolites was determined by dividing the ^13^C_6_ ion signal (and ion signal from ^13^C_12_ and ^13^C_18_ features, when present) for each labeled metabolite feature by the ion signal from the entire pool of the metabolite feature [i.e., ^13^C_6,12,18_ ion signals/(^13^C_6,12,18_ + ^12^C ion signals) for a labeled metabolite feature]. The resulting values were also then converted into a mol% to directly compare the capacity of Phe and Tyr to label each metabolite feature. For example, at the end of the labeling period, 25% of a chlorogenic acid (i.e., caffeoylquinate) pool was isotopically labeled in the ^13^C-Phe-fed leaves, and 4.2% was labeled in ^13^C-Tyr-fed leaves. Then, calculating the relative *de novo* contributions (i.e., mol%) indicated that 86 mol% of leaf chlorogenic acid was derived from ^13^C-Phe, and 13 mol% was derived from ^13^C-Tyr.

**Figure 4:**
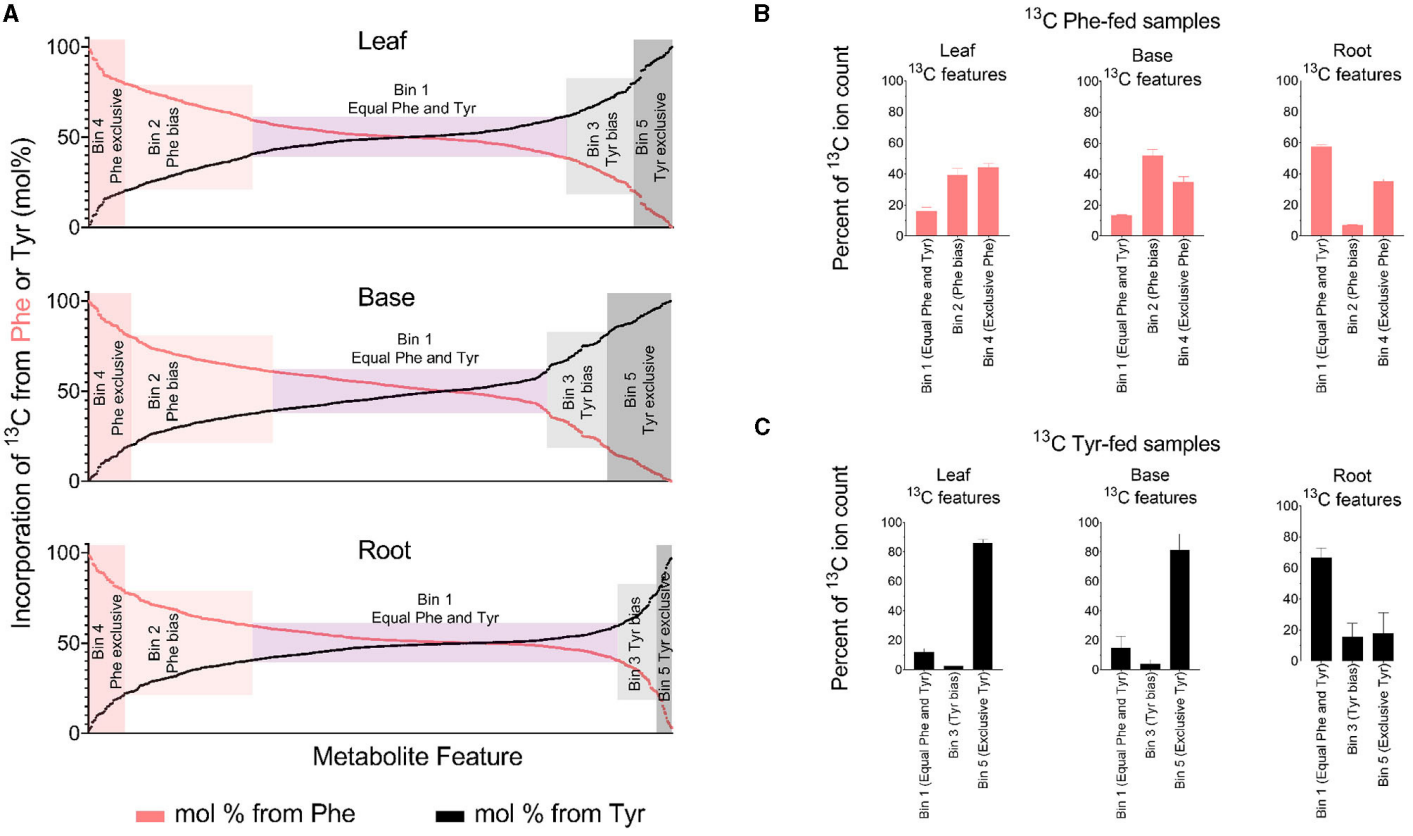
Quantified incorporation of Phe and Tyr into metabolite features. **(A)** Incorporation of ^13^C Phe and ^13^C Tyr, expressed as a mol%. The percent ^13^C incorporation was determined for every peak-pair in ^13^C-fed tissues by dividing the ion counts in the ^13^C-containing features by the sum of the ion counts in its combined ^12^C- and ^13^C-containing features. Each feature was then assigned a mol% based on ^13^C_6_ incorporation from Phe or Tyr. A metabolite feature with 90 mol% labelings from Phe will have a Tyr-derived mol% labeling value of 10, and both of those values are plotted on the graph. For **(A)**, the *t*-test filter that was used to generate Table 1 and **(B,C)** was omitted. Thus, **(A)** includes several MS features that did not meet the *t*-test criteria but had a mol% enrichment value assigning them to one of the five bins (Supplementary Table 2). **(B,C)** Aggregated ion counts from ^13^C Phe **(B)** or ^13^C_6_ Tyr **(C)** into metabolite features in each labeling bin that is described in Table 1. For **(B,C)**, the y-axis refers to the ion count in Phe and Tyr-derived features as a percentage of the ion count in all detected metabolite features comprising the FDM and YDM, respectively.

Metabolite features were sorted into five bins based on mol% enrichment from Phe and Tyr in each tissue and a *t*-test that compared ^13^C incorporation into metabolite features between Phe-fed and Tyr-fed samples (Figure 4, Table 1). Bin 1 contained metabolite features that were labeled at 50 +/– 10 mol% from each of the precursor amino acids (i.e., considered derived equally from both amino acids). Metabolite features in this bin represented the majority of labeled features in each tissue. Bins 2 and 3 contained metabolite features with a mol% enrichment >60% and <80% from Phe (Bin 2) and Tyr (Bin 3), respectively (*p* < 0.05). Metabolite features with a Phe bias were more numerous in all three tissues. Bins 4 and 5 contained metabolite features with an average enrichment of 80 mol% or higher from Phe (Bin 4) and Tyr (Bin 5), respectively (*p* < 0.05), and thus were considered derived exclusively from a single amino acid. The Tyr-derived features were more numerous than the Phe-derived features, but Bins 4 and 5 contained the fewest features.

**Table 1:** Categorizing labeled MS features derived from Phe and Tyr.

**Bin and amino acid bias**	**Percent of labeled MS features in each bin**		
	**Leaf**	**Base**	**Root**
Bin 1: Neither Phe or Tyr bias 50–59 mol % toward Phe or Tyr	44	34	55
Bin 2: 60–80 mol % toward Phe	14	22	16
Bin 3: 60–80 mol % toward Tyr	6	7	4
Bin 4: 80–100 mol % toward Phe	5	7	5
Bin 5: 80–100 mol % toward Tyr	5	10	1

The ^13^C-Tyr and ^13^C-Phe flux into metabolites were estimated by summing the ^13^C ion signal for the respective bins (Figures 4B,C). The total ion count from Tyr-derived metabolites (Bin 5) was several-fold higher than for metabolites derived exclusively from Phe (Bin 4), but only in above-ground tissues. Thus, more of the fed ^13^C-Tyr went into metabolites produced exclusively from that amino acid, despite there being more total PTAL-derived metabolite features produced. Along the same lines, in above-ground tissues, there was a relatively even distribution of the ^13^C signal from metabolites derived exclusively from Phe (Bin 4) and PTAL-derived metabolites with a Phe-bias (Bin 2) as compared to metabolites of approximately equal Phe and Tyr labeling.

**Figure 5:**
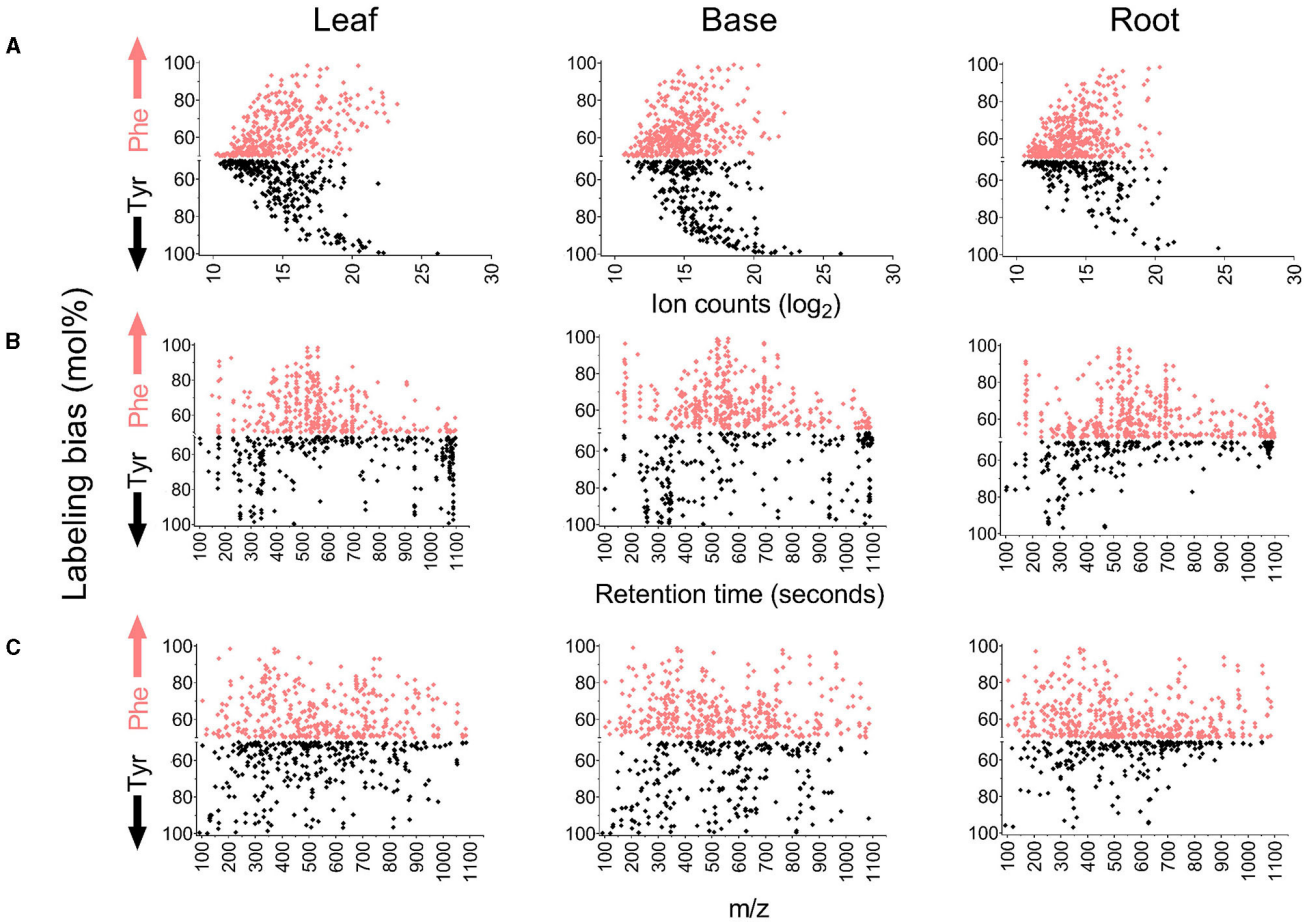
Comparison of properties of metabolite features based on their incorporation of ^13^C Phe or ^13^C Tyr. The y-axis on each graph reports mol% labeling from Phe or Tyr if the bias toward an amino acid was above 50%. Features that fall above the middle axis were more heavily derived from Phe, and features that fall below were more heavily derived from Tyr. For example, an MS feature with a 90 mol% labeling from Phe and 10 mol% labelings from Tyr will be represented on the y-axis by a single point at 90 in the Phe-bias portion of the graph. On the contrary, an MS feature with a 10 mol% labeling from Phe and a 90 mol% labeling from Tyr will be represented by 90 mol% in the Tyr-bias portion of the graph. **(A)** Comparison of labeling bias to the ion counts in each metabolite feature (^12^C-fed samples). **(B)** Comparison of labeling bias to the retention time of metabolite features. **(C)** Comparison of labeling bias to m/z value for metabolite features.

Differences in the abundance, retention time, and m/z for the labeled metabolite features were also compared to their preferred amino acid of origin (Figure 5). Many of the most abundant features in all tissues were predominantly or exclusively derived from either Phe or Tyr (Bins 4 and 5), whereas features derived more evenly from either amino acid were less abundant. Most of the principally Tyr-derived features eluted between 250 and 350 s and approximately 1,100 s, indicating that they are relatively polar or non-polar molecules, respectively. In contrast, most of the features that were more strongly Phe-derived or had a mixed Phe and Tyr origin eluted between 400 and 700 s and are thus intermediate in hydrophobicity. Consistent with this result, it was verified by tandem MS (MS/MS) that the Tyr-derived metabolite dhurrin eluted at 310 s and known flavonoids and hydroxycinnamate esters (HCEs) eluted between 500 and 900 s. The comparison of m/z showed no clear difference based on Tyr or Phe origins.

### A Large Proportion of the YDM Is Derived Independently of PTAL

**Figure 6:**
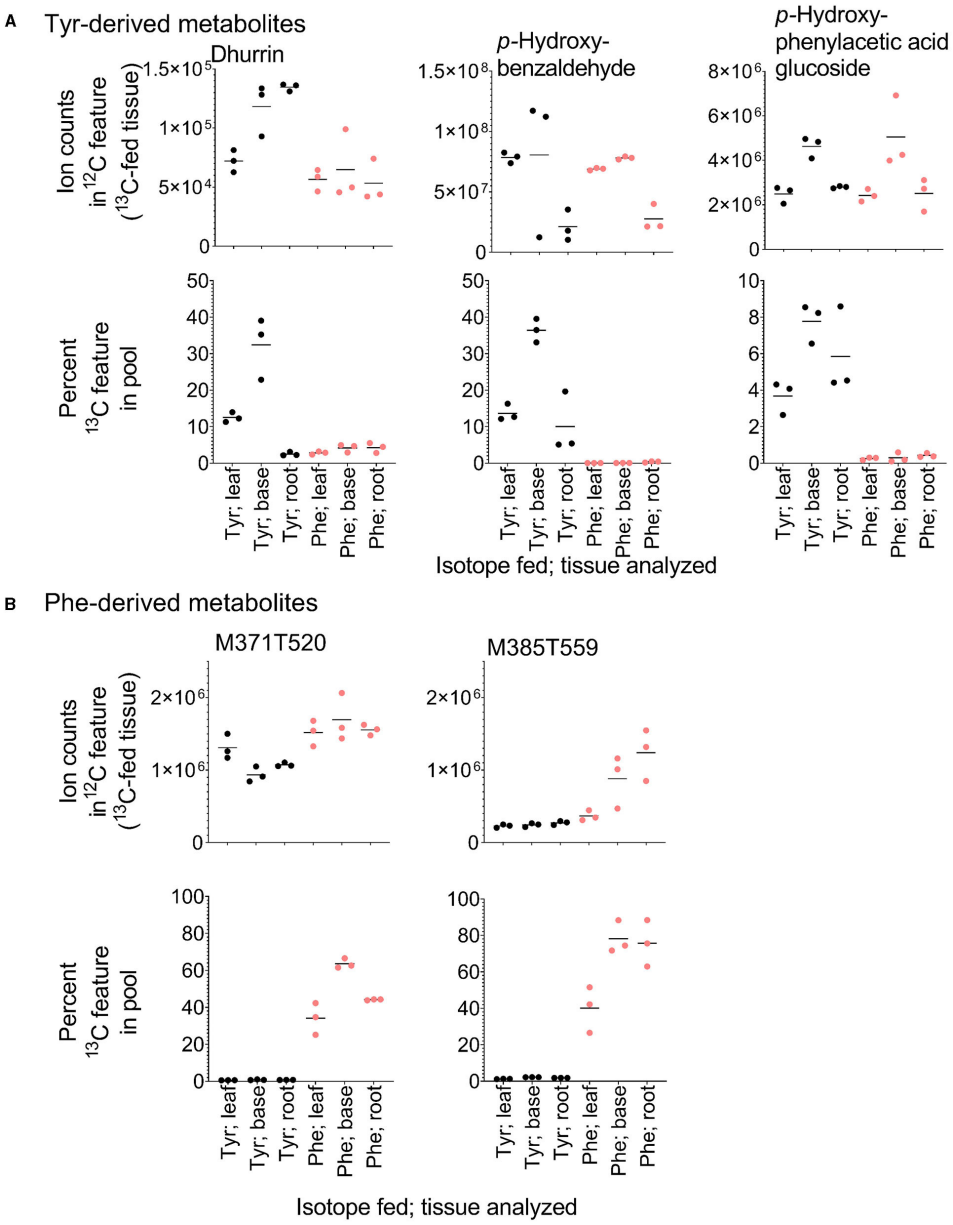
Relative quantification and heavy isotope incorporation into exclusive Tyr- and Phe-derived metabolites from the different tissues and feeding regimes. **(A)** Ion counts into the Tyr-derived features dhurrin, *p-*hydroxybenzaldehyde, and *p-*hydroxyphenylacetic glucoside in tissues fed with each ^13^C isotope (top) and the percent of the dhurrin, *p-*hydroxybenzaldehyde, and *p-*hydroxyphenylacetic glucoside pools that are represented by their respective *de novo* synthesized ^13^C-containing features (bottom). **(B)** Accumulation and labeling into two exclusive Phe-derived features. M371T520 and M385T559 have MS/MS spectra consistent with being esters of benzoic acid and phenylacetic acid, respectively.

Metabolite features derived exclusively from a single amino acid represented only 1–10% of the labeled features in each library (Bins 4 and 5 in Table 1), but constituted a large sink for the ^13^C-Phe and ^13^C-Tyr that was fed to the tissue (Figures 4B,C). Over 80% of ^13^C-Tyr flowed into metabolites that were not produced from Phe, consistent with the fact that sorghum produces large quantities of the cyanogenic glycoside dhurrin (Gleadow and Møller, 2014). In this study, dhurrin was identified in the untargeted MS data as feature M310T325 (which denotes a feature with an m/z M-H value of 310 and retention time of 325 s) and *post-hoc* MS/MS verified this identification and its exclusive Tyr origin (Table 2). ^12^C Dhurrin was detected in all tissues and feeding regimes, but there was a small increase in ^12^C dhurrin in ^13^C Tyr-fed samples and not Phe-fed samples (Figure 6A, top), indicating that the synthesis or catabolism of ^12^C dhurrin was affected by the application of the ^13^C Tyr isotope. *De novo* synthesized ^13^C dhurrin represented between 10 and 40% of the total dhurrin pool (i.e., the proportion of ^13^C dhurrin relative to the sum of ^12^C and ^13^C dhurrin) in the base and leaf portions, but not in the roots (Figure 6A, bottom). The apparent lack of dhurrin biosynthesis in roots has been reported previously (Halkier and Møller, 1989), but some studies have identified dhurrin and its catabolic products, including hydrogen cyanide, in roots (Starr et al., 1984, Adewusi, 1990). There were 39 additional Tyr-derived metabolite features, representing 15 likely parental metabolites, that fell into the exclusive Tyr-derived group (Bin 5), most of which were found only in above-ground tissues. The identities for 11 of these metabolites were also assessed by MS/MS performed subsequent to the initial untargeted analysis. In addition to dhurrin, putative products of dhurrin catabolism were identified, including *p*-hydroxybenzaldehyde (M121T468) and *p*-hydroxyphenylacetic acid glycoside (M313T258) and several other unidentified glycosylated metabolites (Table 2, Figure 6) (Gleadow and Møller, 2014). Although ^13^C dhurrin was not detected in the roots, the presence of labeled catabolic products suggests that they, or ^13^C dhurrin, were transported to the roots from the site of synthesis but, apparently, at a rate too slow to lead to a significant pool of ^13^C dhurrin. Alternatively, the labeled *p*-hydroxybenzaldehyde could originate from transported *p-*hydroxymandelonitrile, which is both a dhurrin biosynthetic intermediate and a breakdown product (Halkier and Møller, 1989).

**Table 2:** Tyr-derived metabolites and their MS/MS fragmentation ratios.

**Feature name**	**^12^C feature m/z**	**^13^C feature m/z**	**Peak pair retention time (seconds)**	Fragment m/z and % of spectrum represented by fragment^*^		**Tentative identification**
				**Phenyl ring-containing fragments**	**Non phenyl ring fragments**	
M121T468	121.0298	127.0496	468	121 (62); 92 (23); 93 (3)	None detected	*p*-hydroxybenzaldehyde
M130T746	130.0299	136.0498	746	131 (35); 130 (5); 102 (13)	None detected	Unknown
M131T1041	131.0376	137.0579	1,041	131 (51); 87 (10); 85 (3)	None detected	Unknown
M162T1088	162.0017	168.0218	1,088	162 (73)	None detected	Unknown
M227T1070	227.0464	233.0663	1,070	227 (42); 168 (12); 167 (6); 142 (2); 141 (15)	66 (12)	Unknown
M263T938	263.0827	269.1026	938	263 (1); 219 (1); 153 (4); 132 (20); 131 (64)		Unknown
M310T325	310.0935	316.1138	325	310 (1); 148 (12); 121 (2); 113 (3)	179 (4); 119 (7); 89 (15); 71 (6); 59 (28)	Dhurrin
M313T258	313.0932	319.1129	258	313 (1); 151 (11); 107 (22); 106 (3)	159 (1); 113 (4); 101 (6); 85 (13); 83 (1)	*p*-hydroxyphenylacetic acid glucoside
M346T312	346.0709	352.0907	312	346 (2); 101 (4)	179 (12); 161 (3); 131 (1); 119 (5); 113(4); 89 (20); 71 (8); 59 (19)	Unknown, non-phenyl ring is consistent with a glucose
M445T311	445.1357	451.1558	311	445 (17); 401 (6)	161 (9); 101 (5); 113 (3); 71 (8); 59 (3)	Unknown, non-phenyl ring is consistent with a glucose
M814T340	814.2629	820.2822	340	814 (1); 472 (2); 683 (5); 503 (1); 341 (23)	179 (9); 161 (23); 113 (17); 89 (4)	Unknown, non-phenyl ring is consistent with a glucose

* *MS/MS was performed after the initial untargeted MS^1^ analysis, and fragmentation spectra were collected from metabolite features identified by PODIUM as being derived from Phe or Tyr*.

A similar analysis identified 16 exclusively Phe-derived metabolite features, representing 10 likely parental metabolites common to all three libraries with an additional metabolite found only in above-ground tissues (Table 3). This list included acetylated phenylalanine and metabolites M371T520 and M385T559 that were tentatively identified as conjugates of benzoic acid and phenylacetic acid, which are molecules that cannot be made from Tyr (Table 3, Figure 6B). Phenylacetic acid has recently been shown to be produced from Phe through a PAL/PTAL-independent pathway involving phenylacetaldoxime (Perez et al., 2021).

**Table 3:** Phe-Derived metabolites and their MS/MS fragmentation ratios.

**Feature name**	**^**12**^C feature m/z**	**^**13**^C feature m/z**	**Peak pair retention time (seconds)**	Fragment m/z and % of spectrum represented by fragment^*^		**Tentative identification**
				**Phenyl ring-containing fragments**	**Phenyl ring-containing fragments**	
M206T561	206.0821	212.1021	561	206 (3); 164 (17); 147 (4); 103 (1); 91 (11)	72 (3); 70 (4); 58 (13)	Acetylated phenylalanine
M371T520	371.0986	377.1187	746	371 (16); 12 (11); 77 (3)	249 (3); 113 (3); 99 (2); 85 (2); 75 (2); 71 (2); 59 (3)	1-*0-*4-benzoyl-3-*O*- glucuronosyl glycerol (Stark et al., 2017)
M385T559	385.1131	391.1327	1041	385 (17); 135 (2); 117 (2); 111 (1); 91 (8)	267 (17); 249 (4); 175 (1); 161 (1); 113 (6); 99 (1); 89 (1)	1-*0-*4-phenylacetyl-3-*O*- glucuronosyl glycerol

* *MS/MS was performed after the initial untargeted MS^1^ analysis, and fragmentation spectra were collected from metabolite features identified by PODIUM as being derived from Phe or Tyr*.

### Phe and Tyr Are Differentially Incorporated Into Common Hydroxycinnamates and Flavonoids

**Figure 7:**
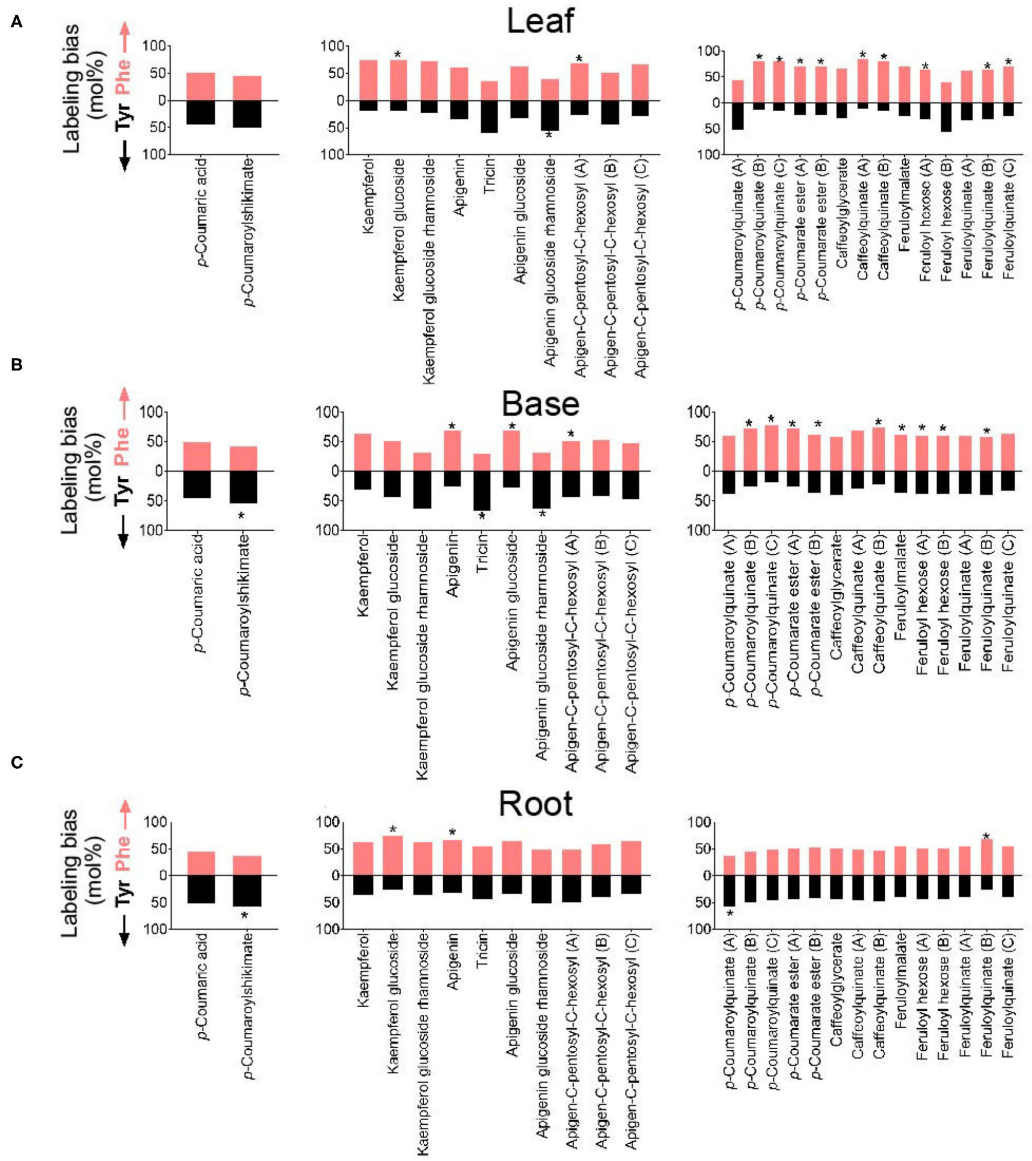
Labeling from ^13^C-Phe or ^13^C-Tyr (expressed as a mol%) into selected phenylpropanoids in **(A)** leaf, **(B)** base, and **(C)** root segments. The identities of metabolites were corroborated by MS/MS (Supplementary Table 1). Monolignol alcohols and other soluble lignin precursors were not detected in these tissues. The metabolite named “coumaroyl ester” has an m/z of 355.06 and contains a Phe-derived *p*-coumarate (m/z 163) portion with an unknown non-Phe derived portion of m/z 209.03. Positional isomers are distinguished by the letter following the name of the metabolite and are ordered in the graph from most polar to least polar. Labeling from Phe is denoted by the upper red bar, whereas labeling from Tyr is denoted by the lower black bars. The stars on bars report whether ^13^C incorporation into the metabolite was significantly different between Phe and Tyr feedings (*n* = 3; *p* < 0.05).

The relative proportions of ^13^C incorporation into putative PTAL-dependent metabolites differed between amino acid treatment and between tissues (Figure 4A). Because the metabolism of Phe and Tyr both produce *p*-coumaric acid, these differences in the incorporation into downstream metabolites suggested more than one *p*-coumaric acid pool exists, with the contributions of Phe and Tyr to these pools not being equivalent. This possibility was examined for individual metabolites by determining label incorporation into *p*-coumaric acid and 25 other abundant HCEs and flavonoids whose structures were predicted by *post-hoc* MS/MS (Figure 7). For *p*-coumaric acid, there was a <10% difference in ^13^C labeling from Tyr and Phe. This measurement represents a tissue-wide average and does not shed light on potentially distinct cellular or subcellular pools. However, downstream of *p-*coumaric acid, the flavonoids kaempferol glucoside, apigenin, apigenin glycoside, and an isomer of an apigenin C-glycoside were enriched in label from ^13^C-Phe. Label from ^13^C-Tyr was enriched in tricin, apigenin glucoside rhamnoside, and an isomer of apigenin C-glycoside. For HCEs, as was seen with *p-*coumaric acid, there was no significant difference in Tyr and Phe incorporation into *p*-coumaroylshikimate and one isomer of *p*-coumaroylquinate in leaves (Figure 7A), although *p*-coumaroylshikimate in the base and root portions were slightly more enriched from Tyr (Figures 7B,C). In contrast, two of the three detected isomers of *p*-coumaroylquinate and most caffeoyl and feruloyl esters were labeled more from Phe than Tyr in the leaf and base portions. In roots, only one isomer of feruloylquinate was more enriched in label from Phe, while the other HCEs were labeled evenly from both amino acids. Taken together, the lack of a consistent labeled Phe-to-Tyr incorporation ratio between metabolites derived through PAL and/or PTAL implies that there is more than one *p-*coumaric acid pool within each tissue and that they are differentially derived from Phe and Tyr.

### The FDM and YDM Include Soluble Phenylpropanoids Affected in *Brown midrib* (*bmr6* and *bmr12*) Mutants of Sorghum

**Figure 8:**
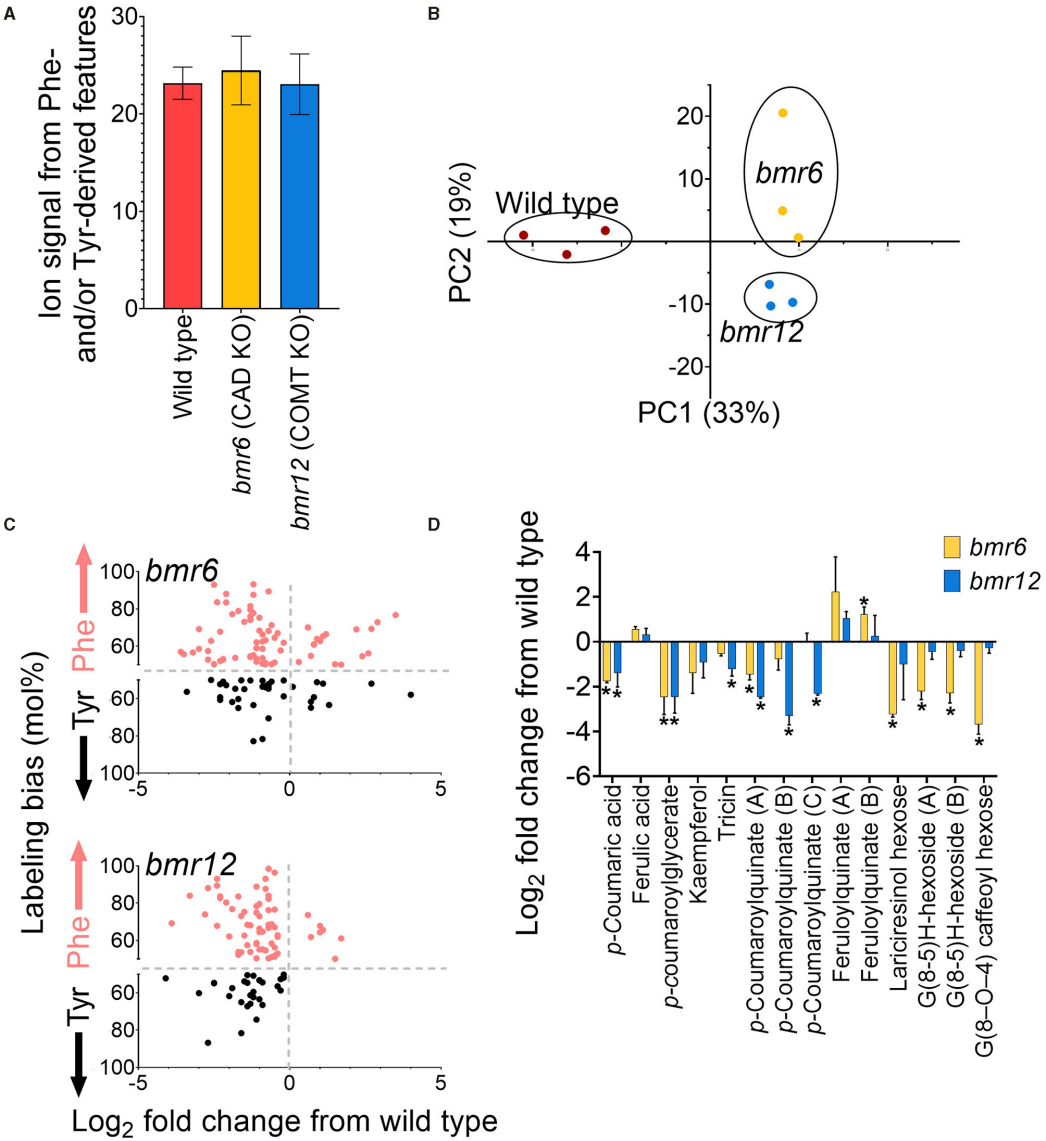
Annotation of Phe- and Tyr-derived metabolite features in expanded leaves of two-week old *bmr* mutants. **(A)** Percentage of MS features *bmr* mutants and the wild-type (BTx623) samples that were retrospectively annotated as Phe- or Tyr-derived. **(B)** PCA analysis of Phe- and Tyr-derived features in wild-type and bmr mutants. **(C)** log_2_ fold change in abundance for MS features is significantly altered in *bmr6* and *bmr12* relative to wild type (*p* < 0.05). The y-axis reports the difference in the incorporation of Tyr and Phe (expressed as a mol%) for the significantly altered metabolite features. This data was extrapolated from the leaf-feeding experiments described in this manuscript. Points above the centerline represent MS features that were more labeled from Phe, and points below the centerline represent MS features that were more labeled from Tyr. **(D)** Log_2_ fold change relative to wild type of selected metabolites in *bmr6* and *bmr12* that were tentatively identified by MS/MS. Stars above points denote statistical significance (*n* = 3; *p* < 0.05).

The library of Tyr and Phe-derived metabolite features was used to characterize the FDM and YDM of two genetically characterized *bmr* mutants, *bmr6* and *bmr12*, which carry loss of function mutations in CAD and COMT, respectively (Palmer et al., 2008). Based on the positions of *bmr6* and *bmr12* in the phenylpropanoid pathway, loss of CAD could negatively affect the production of monolignols and soluble derivatives, such as lignans and neolignans; whereas, loss of COMT activity in *bmr12* could affect the methylation of 5-hydroxyferulic acid, 5-hydroxyconiferaldehyde, and 5-hydroxyconiferyl alcohol into sinapate, sinapaldehyde, and sinapyl alcohol, respectively, and the methylation of flavonoids (Eudes et al., 2017). In total 469 Phe- and Tyr-derived metabolite features were annotated in *bmr* and wild-type leaves, representing 21–28% of the ion counts in those samples (Figure 8A). A PCA using ion abundances of the predicted Phe- and Tyr-derived features separated each genotype along with both components, indicating that the *bmr* mutants have different metabolic phenotypes from each other and those from the wild type (Figure 8B). The total aggregated ion counts for Phe- and Tyr- derived metabolite features in the mutants were not different from those in the wild-type plants.

In total, 163 labeled features were significantly altered in abundance (*p* < 0.05) in one or both mutants, compared to those from the wild type. Of these, most features significantly decreased in one or both mutants relative to those from the wild type (47 in *bmr6* only, 49 in *bmr12* only, and 39 in *bmr6* and *bmr12)*. Those that increased relative to these wild-type plants included 24 metabolite features in *bmr6*, 5 mass features in *bmr12*, and 2 features in both mutants. Zero mass features were significantly affected in both mutants in opposing directions. Most *bmr*-affected features were preferentially derived from Phe in wild type, a result consistent with many other known phenylpropanoids detected through heavy isotope labeling in this study (Figure 8C). Of the MS/MS corroborated metabolites, feruloylquinate hyperaccumulated in *bmr6*, but *p*-coumaric acid, an ester of *p-*coumaroylquinate, kaempferol, and *p*-coumaroylglycerate were reduced in the same mutant (Figure 8D). In *bmr12*, there was also a significant reduction in *p*-coumarate, *p*-coumaroylquinate, *p*-coumaroylglycerate, and the flavonoid tricin (Figure 8D). The reduction in tricin is consistent with a previous report suggesting that the COMT disrupted in *bmr12* also methylates some flavonoids (Eudes et al., 2017). Furthermore, neolignans and lignans are phenylpropanoid dimers produced from monolignols, thus, their biosynthesis is CAD-dependent (Dima et al., 2015). These neolignans and lignans can be distinguished in isotopic labeling data if the metabolite features incorporated more than one labeled phenyl ring and, indeed, the dual-labeled features that negatively affected in *bmr6*, but not in *bmr12*, were metabolites that were tentatively identified as lariciresinol hexose (G (8-8) G) (M521T562), a formate adduct of G (8–*O*−4) caffeoyl hexose (M565T604) (Vanholme et al., 2012), and G(8-5)H-hexoside (M535T598 and M535T623) (Sundin et al., 2014) (Figure 8D).

## Discussion

Although specialized metabolites can be detected and measured by tools such as LC-MS and GC-MS, the identities of most of the mass features collected are not known (Perez De Souza et al., 2017). The PODIUM isotopic labeling protocol and computational pipeline helps solve this by providing precursor-of-origin annotations to metabolites and their features collected by untargeted MS (Simpson et al., 2021). In our previous study, the ability to sort metabolite features into precursor-of-origin groups helped in metabolite identification and the merging of metabolomic data with genomic data to identify genes influencing specific aspects of phenylpropanoid metabolism. Notably, the PODIUM-derived metabolite annotations identified the metabolic consequences of mutating various phenylpropanoid pathway enzymes and uncovered potentially biologically relevant gene-to-metabolite associations through genome-wide association.

Here, PODIUM was applied to identify the soluble FDM and YDM of the reference sorghum genotype BTx623. Despite the emerging importance of sorghum to the bioeconomy, there is considerably less information about its metabolic space, especially compared to *Arabidopsis* and even other monocots, such as rice and maize. The determination of these two sorghum metabolomes in this study sets the stage to identify metabolite-to-gene associations in re-sequenced mutant populations and diversity panels through genome-wide association. In addition, the determination of both the FDM and YDM is particularly relevant in grasses because they possess dual PAL and PTAL activity; thus, this data allows us to compare the relative capacity of sorghum to incorporate each aromatic amino acid into common soluble phenylpropanoids.

The primary focus of this work was to annotate Phe- and Tyr-derived soluble metabolomes of young sorghum plants. Many of the so-called specialized metabolites that confer resistance to biotic and abiotic stresses are contained within this subset of the metabolome. In sorghum, this includes the cyanogenic glycoside dhurrin that is derived from Tyr, and various hydroxycinnamic esters and flavonoid glycosides that are produced by the phenylpropanoid pathway following the deamination of Phe or Tyr. Although our labeling approach captured many specialized metabolites derived from the amino acids, including *p*-coumaric and ferulic acids, the monolignol alcohols that are polymerized into lignin were not detected. Previously, the feeding of ^13^C_6_-Phe to *Arabidopsis* stems resulted in the label incorporation into lignin and the detection of monolignols and free hydroxycinnamic acids (Wang P. et al., 2018). In contrast, the young sorghum plants used in this study were likely not synthesizing secondary cell walls at comparable rates, so the pools of soluble lignin precursors may have been too low to be detected.

The total ion count from Phe-derived metabolites in sorghum was between 25 and 45% of the total ion count, which is a value comparable to what was observed in the Phe-derived soluble metabolome of *Arabidopsis* stems, a plant that does not have PTAL activity (Simpson et al., 2021). Phe-derived metabolite features detected in both species include free hydroxycinnamic acids *p*-coumarate and ferulate, some kaempferol-derived flavonoids, and coniferyl alcohol coupling products. However, for the most part, the two FDMs are very different. The most abundant metabolite features in *Arabidopsis* stems were hydroxycinnamic acids, notably sinapic acid, esterified to glucose and malate, which were not detected in sorghum. In contrast, coumarate, caffeate, and ferulate esters to quinate, and apigenin-containing flavonoids that are produced in sorghum were largely absent from *Arabidopsis* stems.

### Phe and Tyr Contribute Differently to Phenylpropanoid Biosynthesis in *Sorghum*

Phenylalanine/tyrosine ammonia-lyase activity in plants has been known since the initial elucidation of the phenylpropanoid pathway by radioisotope labeling (Brown and Neish, 1956; Neish, 1961; Jangaard, 1974). Dual PAL and TAL activity in plants appears to only exist in grasses, where PTAL activity and the corresponding genes have been functionally characterized in maize (Rösler et al., 1997), *Brachypodium* (Cass et al., 2015; Barros et al., 2016), and sorghum (Jun et al., 2018), among others (Barros and Dixon, 2020). Sequence analysis and mutagenesis indicated that PTALs can use Tyr as a substrate because of a Phe-to-His substitution in the active site of the enzyme (Watts et al., 2006), although additional substitutions may contribute to substrate promiscuity (Jun et al., 2018). Sorghum contains eight PAL isoenzymes, and sequence analysis and *in vitro* characterization identified two of them as PTALs: Sb04g026510 (SbPAL1) and Sb06g022740 (Jun et al., 2018). In this study, precursor labeling identified 668 metabolite features derived from Phe and/or Tyr across three tissues, representing over 20% of the ion signal acquired in each tissue by reverse-phase chromatography and negative-mode ionization. Most labeled metabolite features were labeled from both Phe and Tyr, providing *in planta* evidence that sorghum can use both amino acids to synthesize soluble *p*-coumaric acid-derived phenylpropanoids.

The analysis of the flow of ^13^C into metabolites identified clear differences in the relative incorporation of Phe and Tyr into *p-*coumaric acid-derived phenylpropanoids. The incorporation of labeled substrates is influenced by the size of preexisting precursor pools, the capacity of the substrate to access metabolically active pools, and differences in substrate uptake. The incorporation of Phe and Tyr into metabolites presented here provided an estimation of the capacity of the plant to channel exogenous Phe and Tyr into downstream metabolites. Most labeled metabolite features did not exhibit any precursor-of-origin bias; however, half of the structurally characterized HCEs and flavonoids were more strongly labeled from Phe. This bias was strongest in the two above-ground tissues. These observations are consistent with multiple independent *p*-coumaric acid pools that are differentially utilized to synthesize some PAL and/or PTAL-derived metabolites. Distinct *p-*coumaric acid pools and the idea that PAL and PTAL are responsible for different products were previously suggested after silencing the single PTAL gene of *Brachypodium* (Barros et al., 2016). In the mentioned study, labeled Phe and Tyr were incorporated relatively equally into lignin in wild type Brachypodium. However, in the PTAL RNAi line, syringyl monomers and cell-wall-bound *p*-coumarate were more affected than other lignin or cell wall components. Because PTAL affinity and activity toward Tyr were greater than for Phe, the authors proposed that the TAL functionality of PTAL is preferentially utilized for making those cell wall and lignin components. They also showed that the downregulation of PTAL causes a significant reduction in soluble *p-*coumarate and some flavonoids, whereas ferulic acid and chlorogenic acid increased over 2-fold in that mutant. This data indicated that *Brachypodium* PTAL should be used along with PAL to also synthesize soluble products of the phenylpropanoid pathway, and, similar to the data in sorghum, suggested that PAL and PTAL may have different roles for soluble phenylpropanoid biosynthesis. How Phe and Tyr are metabolized into different *p*-coumaric pools to produce different products could be influenced by several factors including PTAL vs. PAL expression, the kinetic properties of PALs and PTALs, and the compartmentalization of enzymes and substrates within plant cells and tissues. Localized expression differences may contribute significantly to the differences in Phe and Tyr incorporation into phenylpropanoids and/or lignin in *Brachypodium*, where PTAL and PAL genes are co-expressed with different suites of genes (Barros and Dixon, 2020, Barros et al., 2016). Publicly available expression data from sorghum of equivalent age and tissue to the study (accessed from gramene.org) (Davidson et al., 2012; Shakoor et al., 2014; Wang et al., 2018) showed that the PALs with characterized PTAL functionalities (Jun et al., 2018) were more highly expressed than the six other annotated sorghum PAL isozymes. *In vitro* kinetic analysis of *Brachypodium* PTAL revealed a preference for Tyr, although sorghum and maize PTALs had similar activities toward both amino acids (Barros et al., 2016; Jun et al., 2018). Distinct *p-*coumaric acid pools that are utilized differentially could also come about through metabolic channeling between PAL and the ER-bound P450s, such as C4H, that modify phenylpropanoid pathway intermediates (Achnine et al., 2004; Barros and Dixon, 2020). Barros et al. (2016) showed that the lignin from ^13^C-Tyr (and thus requiring its PTAL enzyme) was more sensitive to isotope dilution from exogenously fed *p-*coumaric acid compared with the lignin produced from ^13^C-Phe. This result demonstrates that the endogenous ^13^C-Phe-derived *p*-coumaric acid pool is unable to mix with exogenous *p-*coumaric acid and, thus, points to the physical separation of PAL and PTALs and/or the *p*-coumarate pools to which they contribute (Barros et al., 2016). Therefore, in this model, Tyr would be excluded from PAL-C4H metabolons because it is not a PAL substrate. Alternatively, if Tyr enters a PTAL-C4H metabolon, then the *p*-coumarate generated may be eliminated from the channel because it cannot be acted upon by C4H (Barros et al., 2016; Dixon and Barros, 2019). How these different strategies of *p*-coumarate production would then impact activation by 4-coumarate coenzyme A ligase (4CL) is unclear. Tyr-derived *p*-coumarate acid could also bypass this metabolon *via* the recently discovered cytosolic bifunctional coumarate 3-hydroxyase/ascorbate peroxidase (C3H/APX) that can hydroxylate *p*-coumarate acid directly to caffeic acid (Barros et al., 2019). It was proposed that this pathway may be favored to produce grass-type lignin (i.e., S-type) and the lignin made during stress because of the association of C3H/APX with detoxifying reactive oxygen species (Barros et al., 2019; Barros and Dixon, 2020).

Besides producing *p-*coumaric acid, Phe and Tyr can enter several alternate pathways leading to unique metabolites (Widhalm and Dudareva, 2015; Schenck and Maeda, 2018). Sorghum produces large amounts of the cyanogenic glycoside dhurrin derived only from Tyr (Halkier and Møller, 1989; Gleadow and Møller, 2014) and, accordingly, substantial ^13^C-Tyr incorporation into dhurrin, mass features consistent with dhurrin breakdown products, and other metabolites derived only from Tyr were observed. Metabolites derived exclusively from Phe were present, but ^13^C incorporation into them was considerably less compared to Tyr-only metabolites. Because dhurrin biosynthesis places a high demand on Tyr pools, the observed Phe-labeling bias for many PTAL-derived phenylpropanoids may be due to the reduced availability of Tyr for PTAL. If so, dhurrin accumulating grasses may exhibit a shared pattern of the proportioning of Phe and Tyr into phenylpropanoids.

### Identification of Soluble Phenylpropanoids Affected in *Brown midrib* Mutants

*Brown midrib* mutants (*bmr*) are distinguished by an altered lignin composition and the accumulation of red or brown pigmentation in their leaf and stem vasculature (Sattler et al., 2014). Three *bmr* loci in sorghum encode the known phenylpropanoid pathway enzymes 4CL (*bmr*2) (Saballos et al., 2012), CAD (*bmr*6), and OMT (*bmr12*) (Palmer et al., 2008). The altered and/or reduced lignin phenotypes in *bmr* mutants have been characterized extensively because of their utility in saccharification and forage applications (Sattler et al., 2010). There are contradictory reports on the susceptibility of *bmr* maize and sorghum to diseases, with some studies reporting an increased susceptibility due to impaired cell wall function and others reporting increased resistance, possibly due to the increased levels of phenylpropanoid specialized metabolites (Sattler et al., 2010). Previous work examining soluble hydroxycinnamic acids from mature stems of two different sorghum lines containing *bmr6* or *bmr12* mutations showed increased levels of free ferulic acid, but opposing effects on *p*-coumaric acid where it was decreased in *bmr6* and increased in *bmr12* (Palmer et al., 2008; Tetreault et al., 2020). In this study, a similar effect on *p-*coumarate, and *p-*coumarate and ferulate esters in *bmr6* were noted, but reduced levels of *p-*coumarate esters in *bmr12* were observed as well.

The synthesis of phenylpropanoid dimers, such as neolignans and lignans, requires the production of cinnamyl alcohols by CAD. Accordingly, some of the most negatively affected metabolites in *bmr6*, and not *bmr12*, were dual-labeled metabolites. This result also indicates that the CAD locus affected in *bmr6* is required for lignan synthesis, in addition to its role in lignin deposition. The *bmr6* and *bmr12* mutants of sorghum are defective at the same respective loci as the *cadc, cadd*, and *omt1* mutants of *Arabidopsis* (Fraser and Chapple, 2011). In *Arabidopsis*, the FDMs of *cadc, cadd*, and *omt1* were distinct from each other and those from the wild type, and a similar result was found in sorghum. However, distinct alterations in phenylpropanoid compounds were found in each species. Notably, the loss of *omt1 Arabidopsis* resulted in the accumulation of the glycosylated substrate of the enzyme (5-hydroxyferuloyl hexose), but this metabolite was not detected in sorghum. Both the loss of CADC CADD in *Arabidopsis* and the loss of an equivalent CAD in sorghum caused a reduction in known coniferyl alcohol-derived dimers; however, the loss of these enzymes in *Arabidopsis* was also accounted with a general increase in upstream hydroxycinnamic esters, namely, sinapoyl and feruloyl malate, which was a trend not observed in sorghum.

## Data Availability Statement

The original contributions presented in the study are included in the article/Supplementary Material, further inquiries can be directed to the corresponding author/s.

## Author Contributions

JS, BD, and CC conceived of the project. JS and CC wrote the manuscript. All authors contributed to the conducting of the experiments and the analysis and discussion of the results.

## Funding

This work was supported by the US Department of Energy, Office of Science (BER), Grant DE-SC0020368 (CC and BD), and JS were supported by a United States Department of Agriculture National Institute of Food and Agriculture postdoctoral grant 2018-08121/1019231.

## Conflict of Interest

The authors declare that the research was conducted in the absence of any commercial or financial relationships that could be construed as a potential conflict of interest.

## Publisher's Note

All claims expressed in this article are solely those of the authors and do not necessarily represent those of their affiliated organizations, or those of the publisher, the editors and the reviewers. Any product that may be evaluated in this article, or claim that may be made by its manufacturer, is not guaranteed or endorsed by the publisher.
